# Challenges and Needs in Digital Health Practice and Nursing Education Curricula: Gap Analysis Study

**DOI:** 10.2196/54105

**Published:** 2024-09-13

**Authors:** Karen Livesay, Ruby Walter, Sacha Petersen, Robab Abdolkhani, Lin Zhao, Kerryn Butler-Henderson

**Affiliations:** 1 School of Health and Biomedical Sciences, Royal Melbourne Institute of Technology University, Melbourne, Australia; 2 School of Nursing, Paramedicine, and Healthcare Sciences, Charles Sturt University, Wagga Wagga, Australia

**Keywords:** nursing, digital health, capability, workforce, framework, nursing education, education, digital health practice, clinicians, nurse, nurse graduates, clinical nurses, nurses, nurse educators, teach, teaching, learning, nursing students, student, students

## Abstract

**Background:**

Australian nursing programs aim to introduce students to digital health requirements for practice. However, innovation in digital health is more dynamic than education providers’ ability to respond. It is uncertain whether what is taught and demonstrated in nursing programs meets the needs and expectations of clinicians with regard to the capability of the nurse graduates.

**Objective:**

This study aims to identify gaps in the National Nursing and Midwifery Digital Health Capability Framework , based on the perspectives of clinical nurses, and in nurse educators’ confidence and knowledge to teach. The findings will direct a future co-design process.

**Methods:**

This study triangulated the findings from 2 studies of the Digital Awareness in Simulated Health project and the National Nursing and Midwifery Digital Capability Framework. The first was a qualitative study that considered the experiences of nurses with digital health technologies during the COVID-19 pandemic, and the second was a survey of nurse educators who identified their confidence and knowledge to teach and demonstrate digital health concepts.

**Results:**

The results were categorized by and presented from the perspectives of nurse clinicians, nurse graduates, and nurse educators. Findings were listed against each of the framework capabilities, and omissions from the framework were identified. A series of statements and questions were formulated from the gap analysis to direct a future co-design process with nursing stakeholders to develop a digital health capability curriculum for nurse educators.

**Conclusions:**

Further work to evaluate nursing digital health opportunities for nurse educators is indicated by the gaps identified in this study.

## Introduction

It is widely recognized that digital health technologies have advanced at a rate greater than education about digital health [1]. Indeed, digital health has barely been established in the nursing curriculum, let alone evaluated to match what is needed in the clinical setting [2]. This is a somewhat unusual phenomenon wherein the application of a practice has happened in advance of the evidence that supports it. Digital technologies are constantly evolving to improve access efficiency, safety, and communication, and in turn, the scope of nursing informatics is rapidly evolving at the intersection of health care and information technology [3]. The adoption and optimization of electronic health records (EHRs) continue to be a major focus in nursing informatics, with a growing knowledge base on the importance of user-centered design to improve the usability and functionality of EHRs, ultimately leading to better care and safety [4]. The COVID-19 pandemic accelerated the use of technologies such as EHRs, which led to reported burnout in the nursing profession [5]. Further during the pandemic, there was an increase in the adoption of telehealth technologies, with nurse clinicians playing a crucial role in facilitating telemedicine care, including remote monitoring and consultation [6]. The integration of telehealth into nursing practice has raised the importance of developing telehealth capabilities in nursing graduates and clinicians [7]. However, within health care, digital technologies have been adopted at a pace faster than they can be taught. As a result, digital health technologies are firmly embedded in clinical practice (eg, the electronic medical record, telehealth, and remote monitoring) while they are rarely used or taught in the nursing curriculum [8]. There is consensus [9] that digital health technologies will improve the safety, efficiency, and quality of health care when implemented appropriately. It is essential that nursing graduates are not only skilled in the safe use of these technologies but also aware of the professional, ethical, and potential benefits and risks of these technologies.

The Australian Nursing and Midwifery Accreditation Council Registered Nurse Accreditation standards [10] state that digital health in the curriculum should be informed by the domains of the National Nursing and Midwifery Digital Health Capability Framework [11], at the level of implementation the extent to which these domains are applied is difficult to assess. In order to determine what education is needed at an undergraduate level to provide adequate digital awareness, knowledge, and skills, first it must be understood what is known, what is taught, and what health care expects of graduate nurses.

The Digital Awareness in Simulated Health (DASH) project has, through several successive and iterative phases, examined the current digital health education needs in nursing training in order to address knowledge and skill needs for nurses in the clinical arena. The project aimed to uncover and quantify what is needed in nursing entry to practice degrees regarding digital health through the different lenses of nursing, nurse educators, nurse clinicians, and nurse graduates. The project has been designed based on the Learning Health System model and includes 3 main cyclical phases: practice to data, data to knowledge, and knowledge to practice. The three phases of the project were (1) a systematic review [12]; (2) an interview study of nurses in clinical practice about their digital health use, digital health application, and related challenges during COVID-19, as well as the needs of the graduate nurse [13]; and (3) a survey of nurse educators in Australian universities regarding their knowledge and confidence in teaching aspects of digital health to entry to practice nursing students [14]. The aim of this paper is to present an analysis of the gaps identified through the triangulation of this past research. The process of identifying the gaps was important to assist in understanding ways to develop, plan, and implement educational strategies to bridge the divide and meet the digital health needs within clinical nursing practice [15].

## Methods

### Overview

This gap analysis triangulated the findings from 2 studies of the DASH project. The source of the data is described in each of the papers previously cited in this paper. Although processes for gap analysis can be found in the literature, they represent a wide context of settings and study types that did not directly translate to the anticipated needs of this project. In fact, the learning needs analysis literature provided more direction in developing a process to identify the gaps [16]. In particular, the learning needs process paid attention to the range of contexts of study participants, focused on knowledge skills and attitudes, considered capabilities, and provided an opportunity to reflect on resources [15]. All these elements were important to understand in determining the requirements for nurses entering practice and the capabilities of nurse educators to teach and provide practice opportunities to learners. In addition to the findings from the interviews and survey, the results were triangulated against the National Nursing and Midwifery Digital Capability Framework [11]. ( This framework represents the work of key stakeholders in digital health within Australia and aims to define the digital health knowledge skills and attitudes required for nursing and midwifery practice while providing a basis against which to tailor learning.

The gap analysis was undertaken by the DASH project team. The research team consisted of 4 nurse educators, a digital health academic, and a postdoctoral research fellow. The team collectively reviewed the data from both the interview and survey studies to discern what gaps existed between the interview data, the survey data, and the framework. The review resulted in several questions that formed the basis of the methods, as follows: (1) How knowledgeable and confident are nurse educators to provide the education expected by the framework? (2) What are the barriers encountered by nurse clinicians reflected in the expectations of the framework? (3) What are the nurse clinicians’ expectations of nurse graduates’ digital health capability reflected in the expectations of the framework? (4) What do nurse clinicians identify as challenges that are identified in the framework, but nurse educators lack confidence in teaching? (5) What do nurse clinicians identify as challenges that are missing from the framework? (6) What do nurse educators identify they do not have confidence or knowledge in teaching, but nurse clinicians do not identify it as an adoption challenge and therefore does it need to be in the framework? (7) What do nurse clinicians identify as graduate competencies that are not in the framework? (8) What do nurse clinicians identify as graduate competencies that are in the framework, but educators lack the confidence and knowledge in teaching?

**Figure 1.**
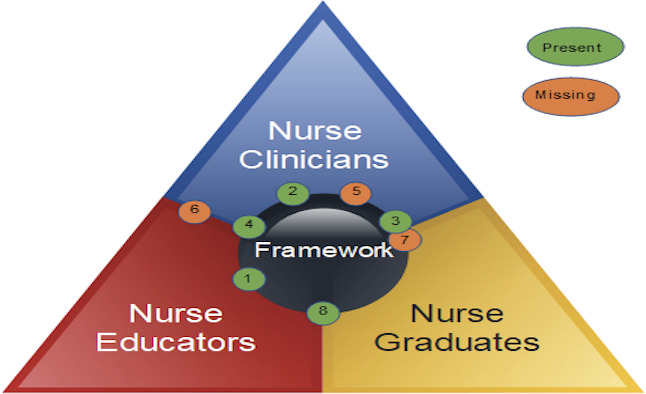
Model of gap analysis.

These questions were mapped in Figure 1 to show how the gaps were identified through the triangulation process, whereby the number represents each of the above questions, and those in green examined items present in the framework as opposed to those in orange that identified items that were missing from the framework.

The procedure to answer each question involved several steps. The first step was to identify the contexts and tools of the nurses who participated in the interview study. There were a range of contexts including nurses in clinical settings, chief nursing information officers, clinical educators, nurse representatives in digital health vendor companies, and nurse representatives in government. The types of digital tools participants had identified using during the COVID-19 pandemic were also identified. The second step involved mapping the barriers to digital health use encountered by those stakeholders during the COVID-19 pandemic to the framework (questions 1, 2, and 3). The survey was devised using concepts from both the framework and the TIGER (Technology Informatics Guiding Education Reform) core nursing informatics competency framework [17], with the method described in a paper by Zhao et al [14]. Survey items that respondents (n=119) had reported “no or minimal knowledge and confidence” were categorized as high where more than 70% of responses chose no or minimum knowledge, medium where 31%‐69% of responses chose no or minimum knowledge, and low where less than 30% of responses indicated no or minimum knowledge. To identify only those items that relate to the Australian framework and remove those that only relate to the TIGER framework, these high and medium items were mapped against the items identified by the nurse educator that appeared in the framework (question 1). The items identified by nurse clinicians as challenges to adoption (question 2) and requirements of the nurse graduate (question 3) in the interviews were mapped to items in the framework. Last, the items identified as challenges to adoption identified by nurse clinicians were mapped against those items that nurse educators identified as lacking the knowledge or confidence to teach (question 4), which resulted in a list of items that need to be taught, but nurse educators lack the knowledge or confidence to teach.

Once these items were mapped to each other and the framework (questions 1-4), the gaps could be identified. This included the items the nurse clinicians identified as challenges that were missing from the framework (question 5); the items nurse educators identified they do not have confidence or knowledge in teaching, but nurse clinicians did not identify as an adoption challenge and raise whether they need to be in the framework (question 6); and the items nurse clinicians identified as nurse graduate competencies that are not in the framework (question 7).

The last part of the triangulation was to identify the items that nurse graduates require to work in health, as identified by nurse clinicians; map them to the framework; and then map them against items that the nurse educators lacked the confidence and knowledge to teach, as identified by the nurse educators themselves (question 8). This resulted in a list of items that nurse graduates need to know, but nurse educators lack the knowledge or confidence to teach. The steps of the procedure linked to the question numbers are summarized in Table 1.

**Table 1.** Procedure for each of the method questions.

Question number	Procedures
Questions 1, 2, and 3	Mapped the qualitative nurse clinician data to the capability statements.
Question 4	Mapped items from the capability statements linked to the nurse clinician’s qualitative data to the nurse educator survey items that scored M^a^ or H^b^.
Question 5	Identified the qualitative nurse clinician data that did not have a competency statement assigned.
Question 6	Compared the H and M items from the nurse educators to the qualitative nurse clinician data where there is no corresponding item in the framework.
Question 7	Mapped the qualitative nurse clinician data about expectations for nurse graduates and identified omissions in the framework.
Question 8	Identified the qualitative nurse clinician data about nurse graduates that mapped to the framework and the nurse educator data that scored an H or an M.

a M: medium.

b H: high.

### Ethical Considerations

Institutional ethics approval (via the Low Risk Committee; reference number: 2022-25054-16817) was provided for each phase of the studies cited. The studies undertaken used informed consent and the ability for participants to opt out in accordance with the ethical standards of the Low Risk Committee and with the Helsinki Declaration.

## Results

The results of the gap analysis were analyzed and presented through the 3 lenses: nurse educator, nurse clinician, and nurse graduate.

### Nurse Educator

Several items were identified by nurse educators that they lacked confidence and knowledge to teach and were related to only the Australian framework (question 1), as summarized in Table 1. No items relating to the domain of “Technology” were identified. The numbers in brackets show the capability statements in the framework the items were mapped against, and the number of participants that reported the lack of knowledge or confidence are reported as medium (M) or high (H), as defined in the *Methods* section.

### Nurse Clinician

There was a strong alignment between the challenges identified by the nurse clinician and the framework across all the domains (question 2; Table 2). Several areas were identified as challenges by nurse clinicians that were not apparent in the framework (question 5). These were classified as either resource or nursing informatics specialist items, as summarized in the last row of Table 2.

There were several areas that nurse clinicians had identified as challenges that were aligned with the framework and nurse educators identified they have a lack of confidence to teach (question 4; summarized in Table 3). When examined at a domain level, every domain except “Technology” contained competency statements that were identified by the nurse clinicians and nurse educators. However, not all statements in these other 4 domains were identified by the nurse clinician and the nurse educators, indicating not all statements are an identified challenge or they are a challenge, but the nurse educators are confident to teach (Table 4). Areas that nurse educators identified they do not have confidence in but were not identified as a challenge by nurse clinicians (question 6; Table 3) were limited and did not include the domains of “Leadership and advocacy” or “Data and information quality.” The “Technology” domain featured the most, however, items related to information systems (eg, radiology, pharmacy, and laboratory) were identified by nurse educators but not by nurse clinicians.

**Table 2.** Alignment of items nurse educators report they lack confidence and knowledge to teach and the framework.

National Nursing and Midwifery Digital Capability Framework domain	Items nurse educators report they lack confidence and knowledge (numbers in brackets represent the capability statement number in the framework; M^a^=31%‐69%, H^b^≥70%)
Digital professionalism	Prescribing and referral rights as it relates to electronic identity (M) (1.2) Cybersecurity and risk management (M) (1.3) Cultural and socioeconomic factors in digital health (M) (1.2) Dynamic consent (H) (1.2) Digital identity (M) (1.2)
Leadership and advocacy	Digital health governance (M) (2.2, 2.3) Patient digital health advocacy (M) (2.1)
Data and information quality	Data, information, and knowledge management (M) (3.2) Processes for reporting quality and safety issues (M) (3.2) Data capture (M) (3.1) Errors in data entry (M) (3.1) Smart phrases (H) (3.1) Smart links (H) (3.1) Quality management (M) (3.2)
Information-enabled care	Information management in clinical research (M) (4.2)

a M: medium.

b H: high.

**Table 3.** Alignment of challenges identified by nurse clinicians and the framework.

National Nursing and Midwifery Digital Capability Framework domain	Challenges identified by nurse clinicians
Digital professionalism	Fear and demotivation in interacting and using a new technology due to lack of preparedness (1.2) Lack of digital health literacy in the senior nursing workforce (1.1, 1.2) Lack of consistent and continuous formal training (1.1, 1.2, 1.3) Lack of time for appropriate training (1.1, 1.2) New technologies led to the emergence of new roles for nurses that required new skill set (1.1, 1.2, 1.3)
Leadership and advocacy	Lack of nurses’ involvement in critical decision making in digital health implementation (2.2) Lack of effective communication among nurses and other stakeholders in using digital health (2.2, 2.3) Lack of communication between managers and ward nurses to understand nurse-specific needs in using digital health (2.2) Current legislations are not applicable nationwide (2.1, 2.3) Lack of legislation to support data transfer between primary and acute care settings (2.1, 2.3) Lack of involvement of external experts in using digital health technologies (2.3)
Data and information quality	Heavy load of digital documentation and nurse shortage to do that (3.2) Lack of access to and use of PROMS^a^ to improve remote management (3.2) The user interface was challenging for immediate clinical actions (3.1) Difficulties in data collection from siloed technologies that are not integrated into the EMRs^b^ (3.1, 3.2) Lack of nurse evaluation of the implemented digital health services (3.3) Lack of feedback and measurements of nurse performance in the digital health systems (3.2, 4.2)
Information-enabled care	Lack of feedback and measurements of nurse performance in the digital health systems (3.2, 4.2)
Technology	Difficulty in communication between nurses and patients in using mobile apps (5.1) Interaction with various screens in telehealth consultations is overwhelming (5.1) Challenges in using interpreters in telemedicine appointments (5.1) Lack of strategies on how to improve access to telemedicine care by culturally and linguistically diverse background communities (5.2) Lack of organizational approach to identify the practice problems that can be solved by a particular technology (5.1, 5.3) Inability of digital health systems to store and analyze a large volume of collected data (5.1) Inability to troubleshoot devices (5.3) Difficulties in reporting errors (5.3)
Items not aligned with framework	Lack of chief nursing informatics officer roles Lack of use of informatics workforce in technology implementations Lack of economists’ perspectives on digital health business models Lack of time to manage the digital content for quality assurance. Lack of funding for continuous evaluation Lack of workforce to know and conduct the evaluation. Interruptions in nurses’ workflows due to lack of computers at bedsides Lack of internet connectivity in distant areas Interoperability challenges among various devices Difficulties in the infrastructure network More cumbersome training in settings that were new to digital health

a PROM: Patient reported outcome measure

b EMR: Electronic medical record

**Table 4.** Comparison of challenges identified by nurse clinicians that align with the framework and areas that nurse educators lack knowledge or confidence to teach.

National Nursing and Midwifery Digital Capability Framework domain	Challenges that nurse educators lack the confidence to teach (numbers in brackets represent the capability statement number in the framework; M^a^=31%‐69%, H^b^≥70%)	Areas nurse educators lack the confidence to teach but were not identified as a challenge by nurse clinicians (numbers in brackets represent the capability statement number in the framework; M=31%‐69%, H≥70%)
Digital professionalism	Prescribing and referral rights as it relates to electronic identity (M) (1.2) Cultural and socioeconomic factors in digital health (M) (1.2) Dynamic consent (H) (1.2) Digital identity (M) (1.2)	Cybersecurity and risk management (M) (1.3)
Leadership and advocacy	Digital health governance (M) (2.2, 2.3) Patient digital health advocacy (M) (2.1)	—^c^
Data and information quality	Data, information, and knowledge management (M) (3.2) Processes for reporting quality and safety issues (M) (3.2) Data capture (M) (3.1) Errors in data entry (M) (3.1) Smart phrases (H) (3.1) Smart links (H) (3.1) Quality management (M) (3.2)	—
Information-enabled care	Information management in clinical research (M) (4.2)	Big data analytics (H) (4.2)
Technology	—	Interoperability (H) (5.1) Troubleshooting (M) (5.3) Clinical decision support systems (M) (5.1) Robotic surgeries (H) (5.1) Blockchain networks (H) (5.1)

a M: medium.

b H: high.

c Not applicable.

### Nurse Graduate

There were a significant number of items the nurse clinicians identified the nurse graduate needs to know that align with the framework (question 3), summarized in . No items were identified in the domains of “Data and information quality” and “Technology.” More importantly, when all the results were triangulated, it identified the items the nurse clinician identified the nurse graduate needs to know, yet the nurse educators lack the knowledge or confidence to teach (question 8; Table 5). Only 1 item was identified—“Students should learn about rules and regulations of data security, privacy, and social media in using digital health (1.2, 1.3).” Clinicians wanted students and graduates to learn about rules and regulations of data security, privacy, and social media in using digital health. This corresponded to the survey item cybersecurity and risk management, which scored 41% for knowledge and 35% for confidence to teach by nurse educators.

**Table 5.** Comparison of items that were identified by nurse clinicians that nurse graduates need to know, that aligned with the framework, and that nurse educators lack knowledge or confidence to teach.

National Nursing and Midwifery Digital Capability Framework domain	Nurse clinician expectations of nurse graduates (numbers in brackets represent the capability statement number in the framework; M^a^=31%‐69%, H^b^≥70%)
Digital professionalism	Students should learn about rules and regulations of data security, privacy, and social media in using digital health (1.2, 1.3)^c^ Students should be taught about nursing’s digital health capabilities before coming into practice (1.2, 4.3) The use of academic EMR^d^ should be a requirement in nursing programs (1.1) Nursing students should learn about digital health systems in more detail than only data entry. For example, about data exchange, security, and analytics (1.1) Students should be taught about real-world digital health challenges in nursing in addition to the theoretical concepts (1.1) Universities should foster digital health training to be responsive to the new generation of technologies (1.1)
Leadership and advocacy	There is a need for investment in the digitally enabled nursing workforce, as they are the only providers in the remote areas of Australia (2.1, 2.2)
Information-enabled care	The concept of a multidisciplinary approach should be embedded in digital health training for nursing students. They need to learn how to interact with internal and external stakeholders (4.1, 4.2) Universities can embed training content about analytics to foster critical thinking and curiosity among nurses about digital health technologies (4.1, 4.2) Students should be taught about nursing’s digital health capabilities before coming into practice. (1.2, 4.3)

a M: medium.

b H: high.

c Identified as a need by nurse clinicians but nurse educators do not have the confidence or knowledge to teach.

d EMR: electronic health record.

## Discussion

### Principal Findings

The triangulation of results from previous studies examined the items identified by nurse clinicians as challenges in digital health adoption in practice, capability needs of nurse graduates, and items nurse educators lack confidence or knowledge to teach (Tables 2-5). These were then mapped to the Australian National Nursing and Midwifery Digital Health Capability Framework [11], and several gaps were identified. These gaps were the main findings of this paper.

A significant difference between the framework and the other reference point, the TIGER study [17] for the development of survey items for nurse educators, is the extent of detail outlining elements within the competency domains in the latter. The international recommendations of the TIGER study identify capabilities associated with recognized nursing informatics roles, which the Australian framework does not identify. The framework with 5 domains and limited detail is open to interpretation as to which domain an area of digital knowledge would best be mapped to. The nurse researchers in the study required advice from digital health academics on the team to clarify the best fit when undertaking this mapping, indicating nurse educators may not be able to implement the framework within their own curriculum without digital health expertise guidance.

The alignment between the challenges identified by the nurse clinician and the framework (Table 4) confirmed the necessity of those items in the framework. Further to the criticism listed above about the framework being open to interpretation is that users of the framework are unable to gauge the depth of knowledge required to achieve capability, as these will vary depending on the statement and the functions of the Clinical Nurse. These items, identified by nurse clinicians (Table 3), can guide users of the framework on the depth of knowledge required for nurse graduates and nurse clinicians for these particular items. The small number of items identified by both the nurse educator and the nurse clinicians, and in particular the lack of items related to the domain “Technology” (Table 3, last row), should not be assumed to mean the other statements are not relevant to practice and should be removed from the framework. Instead, this finding can guide the depth of knowledge required for the nurse graduate. Some areas related to technology are subject to their own programs of graduate study, for example, cybersecurity, and nurses entering practice in a field like this would require a high level of knowledge. The identification of different information systems by nurse educators (Table 4), which were not identified as a challenge by nurse clinicians, may be related to the vendor nature of these information systems. Training is generally provided by the vendor for tendered information system adoption, whereas noninformation system capabilities that a graduate nurse needs to have to be able to work safely and responsibly with technology or data needs to be developed either through graduate training or on the job.

Nurse clinician expectations of nurse graduates did not map to the framework, with the exception of 1 item (Table 5). The significance of this is speculative but likely relates to the questions used in the interview study [13]. As no reference was made to the framework, the responses were not provided that specifically addressed it. Additionally, the questions were broad, and the answers provided also lacked detail. In general, nurse clinician responses generally focussed on global capabilities, such as using an electronic medical record, rather than detailed responses. Further, the framework did not identify several areas identified by nurse clinicians related to resources (Table 3). These resources could be categorized as either physical or human. For example, clinicians spoke about specialist digital or informatics roles that provide support for digital health initiatives, which is not addressed in the framework. Additionally, nurse clinicians spoke about being able to access the correct tools appropriate for the task, including having access to secure internet services. While Standard 5.1 covers recommending appropriate digital technologies and staff and consumers being able to use these where available, the framework does not address a requirement for availability. The growth of digital capacity among nurses in practice will continue to be hampered by under-resourcing. Nurse clinicians called out expertise and human support by other nurses as a barrier in practice. These items cannot be addressed educationally and therefore were not included in the survey of nurse educators (Table 2). Nonetheless, the silence in the framework may result in a missed opportunity to recognize and support specialist nurse practice in digital health. Booth et al [8] suggest support from all levels of nurse leadership to invest in resources and champion and support nurses in their practice and research. The complexity of health environments and the rapid rate of change and development in health care, inevitably result in nurses with a wide range of knowledge and confidence in digital technology use and complex differences in demands and access to digital tools [8,18].

There are several implications for both education and practice. While this gap analysis has identified there was only 1 item that the nurse clinicians identified is required for the nurse graduate but the nurse educator lacked the knowledge or confidence to teach (“Students should learn about rules and regulations of data security, privacy, and social media in using digital health”), there are several items across the domains that nurse educators reported they will be unable to teach but are important skills or knowledge for practice. Given the nurse researchers in this project needed to consult with the digital health experts on the team to interpret elements of the framework highlighted this challenge. It implies that digital health expertise may be required for graduate training providers to meet the Australian Nursing and Midwifery Accreditation Council Registered Nurse Accreditation standards requirements related to digital health. The practicality is most graduate training providers do not have this resource available and it was not the intention of the framework. Nurse educators need to be upskilled in digital health, so they have the confidence and knowledge to design and deliver the necessary digital health curriculum.

The nurse clinicians identified practice barriers relevant to their own context during the COVID-19 pandemic. Nazeha et al [18] recommend that frameworks for digital health be updated regularly in line with innovation. However, the applicability of digital tools and technologies is never going to be universal or static and will always be context-specific. Therefore, considering those items that nurse clinicians did not identify as barriers and nurse educators lacked confidence or knowledge to teach, may be as simple as being out of context of experience for those practitioners and educators. For example, robotic surgery is a very defined digital technology, whereas EHRs are a generic concept. Educators may have an oversight or awareness of a specific digital tool but have answered negatively in the survey as their knowledge is global rather than specific and would not be sufficient to teach or demonstrate to learners.

The items that educators lacked confidence or knowledge to teach should be examined for their value in a crowded, busy curriculum. Nurses, as lifelong learners, continue to develop, and many enter and exit the education system more than once across their careers [19]. Postgraduate study requirements may include specialist knowledge not apparent for entry to practice minimum standards. Risling [20] predicted exponential increases in technology use in the coming decade and warned that nurse educators need to lead the evolution in practice and education. Changes to curriculum, while challenging, will need to be carefully considered for their worth at the same time as recognizing the rapidity of change likely to be required.

The findings from the interview and survey studies informing this analysis may have been influenced by the mixed progress of digital health roll out nationally. A co-design process to develop an educational intervention will be undertaken as the next stage of this DASH research project. The following questions and statements will form the basis for discussion with a broader panel to validate the findings of this analysis and develop strategies to overcome the challenges and weaknesses in clinical practice and educational delivery settings:

- Digital health expertise and guidance are required for nurse educators to develop curricula in digital health.
- The framework requires augmentation to describe the depth of knowledge and experience.
- Should specific technologies be inherent within the framework?
- How should differences in experience, exposure, and resources related to digital health be addressed in nursing education?
- Which of the items that educators lacked knowledge or confidence to teach should be addressed in the curriculum for nurse educators teaching entry-to-practice nursing courses?

### Limitations

In the interview study, we did not ask what skills students were noted to have in digital health, rather, the approach was aspirational for what clinicians desired. The extent to which those aspirations for students are achieved is unknown. It is suggested that a further study investigating the actual capability of nurse graduates in digital health be undertaken.

### Conclusion

This analysis took the outputs of 2 studies investigating the digital health perspectives of nurses in practice environments and their expectations of graduates’ digital health capabilities, with the second paper investigating the capabilities of nurse educators to teach and practice digital health, and mapped these findings with the National Nursing and Midwifery Digital Capability Framework. The outcomes of this analysis will inform a co-design process to create a curriculum for nurse educators to uplift capability in teaching and simulation for entry-to-practice nursing programs in Australia. A series of 8 questions directed the process to triangulate the findings and identify which factors were and were not included in the framework. A series of statements and questions were then formed from the analysis as recommendations to direct the co-design phase of this national research project.

## Abbreviations

DASH: Digital Awareness in Simulated Health
EHR: electronic health record
TIGER: Technology Informatics Guiding Education Reform
